# Dual functions of Insig proteins in cholesterol homeostasis

**DOI:** 10.1186/1476-511X-11-173

**Published:** 2012-12-18

**Authors:** Xiao-Ying Dong, Sheng-Qiu Tang, Jin-Ding Chen

**Affiliations:** 1 College of Veterinary Medicine, South China Agricultural University, No.483 Wu Shan Road, Tian He District, Guangzhou, 510642, China; 2 College of Yingdong Agricultural Science and Engineering, Shaoguan University, Daxue Avenue, Zhenjiang District, Shaoguan, 512005, China

**Keywords:** Cholesterol homeostasis, HMG-CoA reductase, Insigs, Mechanism, SCAP, SREBP

## Abstract

The molecular mechanism of how cells maintain cholesterol homeostasis has become clearer for the understanding of complicated association between sterol regulatory element-binding proteins (SREBPs), SREBP cleavage-activating protein (SCAP), 3-hydroxy-3-methylglutaryl coenzyme A reductase (HMG-CoA reductase) and Insuin induced-genes (Insigs). The pioneering researches suggested that SREBP activated the transcription of genes encoding HMG-CoA reductase and all of the other enzymes involved in the synthesis of cholesterol and lipids. However, SREBPs can not exert their activities alone, they must form a complex with another protein, SCAP in the endoplasmic reticulum (ER) and translocate to Golgi. Insigs are sensors and mediators that regulate cholesterol homeostasis through binding to SCAP and HMG-CoA reductase in diverse tissues such as adipose tissue and liver, as well as the cultured cells. In this article, we aim to review on the dual functions of Insig protein family in cholesterol homeostasis.

## Introduction

Cholesterol is a vital component of cell membranes without which the cell can not function, and it is also the precursor to all steroid hormones, bile acids, and oxysterols, which by themselves are important regulatory molecules in many metabolic pathways. However, its over-accumulation can clog arteries and cause heart disease [1]. So, how to keep the balance of cholesterol metabolism is very important and it has driven many researchers to carry out comprehensive investigations. And the understanding of cholesterol regulation has come a long way from the initial recognition of cholesterol feedback inhibition of its rate-limiting synthetic enzyme, HMG-CoA reductase through the role of lipoproteins in maintaining plasma cholesterol levels, to the recent discovery of regulation of cholesterol synthesis via SREBP pathways.

Four members of the SREBP family, SREBP-1a, SREBP-1c, SREBP-2 and SREBP-2gc, have been identified [2-5]. SREBPs are a family of transcription factors that have independently been characterized as mediators of cellular cholesterol homeostasis [6,7] and as regulators of fatty acid biosynthesis and uptake [8-10]. It has been discovered that SREBP-1a and SREBP-1c control over fatty acid synthesis [11-13], whereas SREBP-2 favors cholesterol synthesis [14]. SREBP-1a induces enzymes for fatty acid elongation and desaturation. Therefore, over-expression of SREBP-1a in adult rats results mainly in over-stimulation of fatty acid synthesis [15-17]. SREBP-1c is involved in the regulation of adipogenesis [18,19], but *in vitro* it does not stimulate cholesterol synthesis [20,21]. SREBP-1c also triggers the expression of genes of enzymes required for fatty acid elongation [17], and of glycerol 3-phosphate acyltransferase required for triglyceride and phospholipid synthesis [22]. SREBP-2 mainly controls the expression of genes involved in cholesterologenesis [23,24], its over-expression can trigger all 12 enzymes of the cholesterol biosynthetic pathway, and induce the cholesterol synthesis markedly [14]. SREBP-2gc, a shortened version of the N-terminal portion of SREBP-2, is not subject to feedback control by sterols, and its expression remains restricted to male germ cells, where it regulates the transcription of spermatogenic genes in a cell- and stage-specific manner [4,5]. Considered together, available experimental data indicate that SREBPs especially SREBP-2 mediate cholesterol metabolism, but additional factors are required to activate this SREBP pathways. As mentioned firstly, SCAP is a potent activator of SREBP pathways in cholesterol synthesis. In the presence of high cellular sterol levels, SCAP confines SREBP to the ER. With low sterol concentrations, SCAP escorts SREBP from ER to Golgi where SREBP undergoes two proteolytic cleavage steps to release the mature, biologically active transcription factor, nuclear SREBP (nSREBP). Then nSREBP translocates to the nucleus and binds to sterol response elements (SRE) in the promoter/enhancer regions of target genes [20].

More recently, Insigs, including Insig-1 and Insig-2, two ER members of Insig proteins family, are discovered and regarded as crucial roles in cholesterol metabolism [25,26]. Insigs are shown to cooperate with sterols to inhibit exit of the SCAP/SREBP complex from the ER to the Golgi [27-29]. Moreover, Insigs negatively regulates HMG-COA reductase transcription by suppressing activation of the ER membrane bound transcription factor SREBP [30,31]. Another investigation further demonstrates that in Insig-1 and Insig-2 knockout mice liver, cholesterol and triglycerides are over-accumulated [32]. Thereby, Insigs appear to mediate reaction of cholesterol synthesis through their sterol-dependent binding to the SCAP and HMG-CoA reductase proteins. For their binding activities, Insigs play an important role in cholesterol homeostasis in different tissues and in cultured animal cells, and make the mechanism of cholesterol homeostasis more transparent. We ever published a review article to discuss the discovery, expression, structure, regulation and gene polymorphisms of Insigs, and their deficiency with diseases [33]. In this review, we mainly focus on how Insigs exert their dual functions in cholesterol homeostasis and what is the molecular mechanism in the cholesterol regulatory system.

### Insigs and their dual functions in cholesterol Homeostasis

#### Special regions in SCAP, HMG-CoA reductase and Insigs

Mammalian cell cholesterol levels are controlled by coordinated regulation of the proteins SCAP and HMG-CoA reductase [34], and these functions are activated through the bindings to Insigs [26,28]. The results in mutant CHO cells demonstrate an absolute requirement for Insig proteins in the regulatory system that mediates lipid homeostasis in animal cells [35]. Both Insig-binding proteins have a similar organization: an N-terminal polytopic membrane domain containing eight membrane-spanning segments [36], and a long hydrophilic C-terminal extension that projects into the cytosol [36,37]. In SCAP, this extension is composed of multiple WD-repeat domains that form propeller-like structures binding to SREBPs [38,39] and also coat proteins clustering SCAP/SREBP complexes into CopII vesicles that bud from the ER [40]. In HMG-CoA reductase, the globular cytosolic domain contains all of the catalytic activities of the enzyme [41,42]. In both cases, the polytopic membrane domain is the site of sterol regulation [43,44], and the C terminal extensions can be deleted without abolishing sterol-dependent binding to Insigs [28,30]. The Insig binding to SCAP and reductase requires the tetrapeptide sequence YIYF in the second membrane-spanning helix of reductase and in the sterol-sensing domain (SSD) of SCAP [45-47]. SCAP, acting through its SSD, mediates feedback regulation of cholesterol synthesis. Point mutations within the SSD of SCAP and reductase prevent their association with Insigs, thereby abrogating sterol-mediated ER retention of SCAP-SREBP and sterol-induced ubiquitination/degradation of reductase [45,48,49].

The sterol response elements, 380 base pairs upstream of the transcriptional start site in Insig-1 [50] and a 350 bp region upstream of the transcription start site in human liver Insig-2 gene were found to be regulated by transcriptionally active SREBP [51]. Many other studies identified that crucial amino acid residues in Insig-1 and Insig-2 were required for their function in binding to SCAP and HMG-CoA reductase in mammalian cells. Gong et al. (2006) reported that the conserved Asp-205 in Insig-1 and the corresponding Asp-149 in Insig-2, which abuts the fourth transmembrane helix at the cytosolic side of the ER membrane, was essential for cholesterol homeostasis [52]. The intramembrane glycine-39 localizes to the first membrane-spanning segment of Insig-2 and Insig-1 was regarded as a key residue for normal sterol regulation in animal cells [53]. When these amino acids were mutated, the mutant Insig proteins lost the ability to suppress the cleavage of SCAP and to accelerate sterol-stimulated degradation of HMG-COA reductase.

#### Insigs bind to SCAP

The synthesis of cholesterol and other membrane lipids in mammalian cells is regulated by the controlled transport of SREBPs from the ER to the Golgi complex. SREBPs are membrane bound transcription factors that activate more than a score of genes encoding enzymes of lipid synthesis. Immediately after their translation on ER membranes, SREBPs bind to SCAP, a polytopic membrane protein that serves both an escort for SREBPs [38,39] and a sensor of sterols [46]. Insig proteins are essential elements of this SREBP pathway, which not only reduce the concentration of cholesterol needed *in vitro* to produce the conformational change in SCAP, but also enhance the conformational change in SCAP that occurs upon addition of certain cationic amphiphiles, such as chlorpromazine, trifluoperazine, and imipramine, which mimic the effect of cholesterol [54]. The conformational change involves arginine-503, which resides in loop6 between membrane-spanning helices 6 and 7. Therefore, sterols bind directly to SCAP and alter the conformation of loop 6, thereby causing SCAP to bind to Insig [54,55]. Insigs exert their functions through the binding to SCAP, the results in mutant CHO cells demonstrate it. When SCAP bears mutations, its binding to Insig is disrupted, and ER retention does not occur [28,56], SREBPs cannot move to the Golgi, cholesterol synthesis does not occur, and the cells require exogenous cholesterol for growth [57,58].

**Figure 1:**
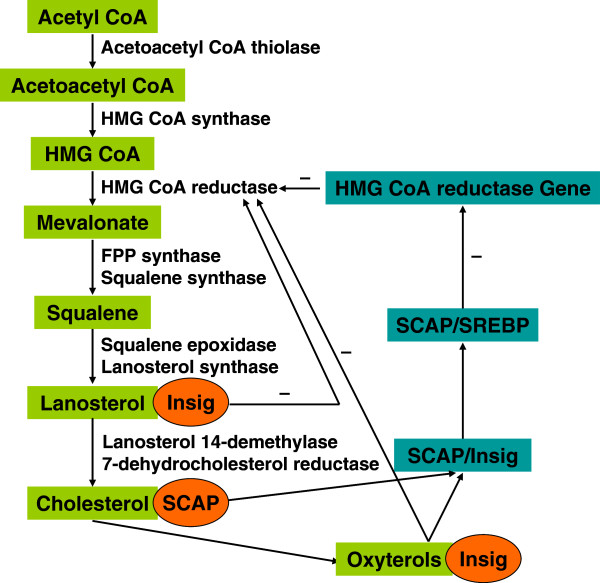
**Feedback control of cholesterol synthesis through binding to Insigs.** HMG-CoA reductase is subject to feedback inhibition by cholesterol, oxysterols and lanosterol. Cholesterol binding to SCAP causes a conformational change that induces SCAP to bind to Insigs. So, cholesterol inhibits reductase activity by suppressing the activation of SREBPs. Oxysterols binding to Insigs inhibit reductase by accelerating its degradation and by suppressing the activation of SREBPs. Lanosterol down-regulates reductase solely by accelerating degradation of the enzyme.

Further studies give us a more apparent molecular metabolism for SREBP pathway *in vitro* and *in vivo*. Co-immunoprecipitation and native gel electrophoresis experiments demonstrate that SCAP binds to Insigs only in the presence of sterols, either cholesterol or 25-HC [28,59]. As illustrated in Figure 1, when sterols over-accumulate in cells, SCAP is retained in the ER, SREBP cannot be processed, and the synthesis of cholesterol declines [60,61], because Insig binds to the membrane domain of SCAP, thereby retaining the SREBP-SCAP complex in the ER and blocking the proteolytic activation of SREBP [31]. In the absence of sterols, SCAP does not interact with Insig proteins. As a result, SCAP escorts the SREBPs into budding vesicles that reach the Golgi complex, where it is cleaved and thus activates to transcribe genes encoding cholesterol biosynthetic enzymes and the LDL receptor [27,62]. Studies in tissue culture show that mutant SCAP is resistant to inhibition by sterols. Cells that express a single copy of this mutant gene overproduce cholesterol [48]. To learn whether SCAP performs the same function in liver as in cultured cells, Korn et al. (1998) used transgenic mice that expressed a mutant liver SCAP with a single amino acid substitution in the SSD and found that levels of nSREBP-1 and nSREBP-2 were elevated to induce the increase of the expression of all SREBP target genes thus stimulating cholesterol and fatty acid synthesis and causing a marked accumulation of hepatic cholesterol and triglycerides [63]. These models *in vivo* and *in vitro* provide strong evidence that Insigs’ binding to SCAP and SCAP activity is normally under partial inhibition by endogenous sterols. Elucidating this mechanism will be fundamental to understand the molecular basis of cholesterol homeostasis.

#### Insigs bind to HMG-CoA reductase

**Figure 2:**
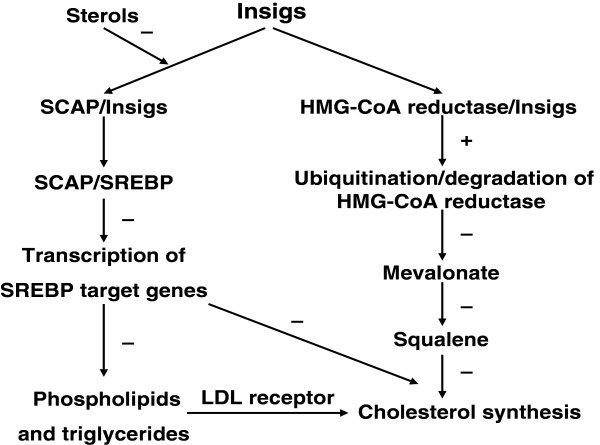
**Dual functions of Insigs in cholesterol metabolism.** Insig proteins exert their dual functions in cholesterol metabolism through binding to SCAP or HMG-CoA reductase. When sterols are over-accumulation, Insigs’ binding to SCAP prevents delivery of SCAP/SREBP complex to the Golgi. For this reason, transcriptional genes are needed for uptake and synthesis of cholesterol, fatty acids, phospholipids and triglycerides decline. Insigs’ binding to HMG-CoA reductase leads to the ubiquitination/degradation of the reductase. The degradation of HMG-CoA reductase inhibits cholesterol synthesis.

HMG-CoA reductase catalyzes the rate-limiting step in the synthesis of cholesterol [64] (Figure 2). A key mechanism for maintaining cholesterol homeostasis in mammalian cells involves modulating the stability of HMG-CoA reductase [37,41,43] through a complex, multivalent regulatory system mediated by mevalonate-derived products [64,65]. Part of this regulatory system involves sterol-regulated ubiquitination and degradation [66-68], which is mediated by the reductase membrane domain and leads to ER-associated degradation of the enzyme [30].

The ER enzyme HMG-CoA reductase produces mevalonate, which is converted to sterols and to other products, including geranylgeraniol groups attached to proteins [64]. Similar to other proteins of the mevalonate pathway [69], HMG-CoA reductase is controlled also at the transcriptional level by SREBP-2, which binds to the promoter of the HMG-CoA reductase gene and activates the transcription when demands for mevalonate-derived products increase [70]. Sever et al. also take investigations in animal cells to demonstrate that Insig-1 and Insig-2 accelerate degradation of reductase. In mutant cells lacking Insig-1, sterols fail to stimulate sterol-dependent ubiquitination and degradation of reductase [30,71]. Through the use of RNA interference, the combined knockdown of Insig-1 and Insig-2 abolishes sterol-induced ubiquitination of endogenous reductase, an obligatory reaction in accelerated degradation of the enzyme [45]. And sterol-accelerated degradation of reductase is inhibited when reductase is over-expressed in CHO cells by transfection, while over-expression of Insig-1 or Insig-2 can restore degradation of reductase [30]. Taken the results together, in animal cells, Insigs act as a central regulator of cellular cholesterol homeostasis by controlling activity of HMG-CoA reductase in cholesterol synthesis. For completing their functions, reductase and Insigs must dislocate to the cytosol and form a tightly bound complex. Dislocation of HMG-COA reductase and Insigs requires metabolic energy and involves the AAA-ATPase p97/VCP [72]. Then HMG-CoA reductase binding to Insigs leads to the ubiquitination of reductase by an Insig-bound ubiquitin ligase, gp78 [68]. RNA interference studies reveal that the degradation of HMG-CoA reductase requires the Drosophila Hrd1 ubiquitin ligase and several other proteins in Drosophila S2 cells [73]. Insigs accelerate the degradation of HMG-COA reductase and suppress their transcriptions through the SREBP-SCAP pathway [27]. However, in the fission yeast, homologs of Insig, HMG-COA reductase, SREBP and SCAP, called ins1, hmg1, sre1, and scp1, Ins1 is dedicated to regulation of Hmg1, but not the Sre1-Scp1 pathway. Insig regulates sterol synthesis by a different mechanism than in mammalian cells [74].

#### The ratio of Insig to its two targets is a crucial requirement for cholesterol metabolism

Studies have shown that the regulatory actions of Insigs in cholesterol metabolism are critically dependent on the ratios of Insig proteins to their targets SCAP and reductase [28,30]. Over-expression of SCAP or reductase through transfection saturates endogenous Insigs, and regulation no longer occurs unless Insigs are also over-expressed in mutant CHO cell line [49]. Defective regulation also occurs when SCAP is over-expressed to such a high level that Insig becomes saturated. Thus, a high ratio of SCAP to Insig diminishes sterol sensitivity of SREBP processing. Conversely, as Insig levels rise, SREBP processing is inhibited by lower concentrations of sterols [28]. The results above all highlight the importance of SCAP-Insig ratios in normal sterol-regulated processing of SREBPs in cultured cells.

#### Insigs are a key point in product feedback inhibition of cholesterol synthesis

End-product feedback inhibition of cholesterol synthesis was first demonstrated in living animals by Schoenheimer 72 years ago [75]. For 30 years, using *in vitro* and *in vivo* assays, scientists have known that the cholesterol regulatory system is controlled not only by the end product cholesterol, but also by oxysterols [76,77], delta- and gamma-tocotrienols [78], and methylated sterols such as lanosterols [79]. While the mechanism is unknown, the current results provide a clear understanding of how cells coordinate this function (Figure 1). Because Cholesterol and oxysterols both induce the SCAP Insig interaction [59], thereby inhibiting the transport of SREBPs from the endoplasmic reticulum to the Golgi [80], and blocking the cholesterol synthesis [29,61]. But they do it by two different mechanisms: 1) cholesterol acts by binding to SCAP, thereby causing a conformational change that induces SCAP to bind to Insig [59,81], the conformational change can be monitored by a change in the tryptic cleavage pattern of SCAP [82]; 2) oxysterols act by binding to Insigs, causing Insigs to bind to SCAP [83]. And Insigs are regarded as oxysterol-binding proteins, explaining the long-known ability of oxysterols to inhibit cholesterol synthesis in animal cells [83]. However, Lanosterol which has been implicated as a rate-limiting step in cholesterol synthesis [84,85], delta- and gamma-tocotrienols [78] accelerate ubiquitination and degradation of HMG-COA reductase without effect on ER to Golgi transport of SCAP/SREBP complex, which contributes to feedback inhibition of synthesis of cholesterol and non-sterol isoprenoids, and this activity requires Insig-1 and Insig-2 [79]. Sterols also stimulate degradation of HMG-COA reductase in Drosophila S2 cells, but only when mammalian Insig-1 or Insig-2 is co-expressed [73]. We conclude that lanosterol, delta- and gamma-tocotrienols inhibit their own synthesis through the down-regulation of reductase, thus inhibiting the sterol pathway and the specificity of lanosterol for reductase may permit the identification of proteins selectively recruited to the reductase-Insig complex. So, Insigs play a critical role in regulating cholesterol concentrations through end-product, oxysterols and lanosterols feedback inhibition in the cells, and avoiding the toxic over-accumulation of cholesterol.

## Insigs are associated with hypercholesterolaemia

Hypercholesterolaemia is a syndrome for the over-accumulation of cholesterol whose excessive amounts in cells can destroy membrane function, precipitate as crystals which will kill the cell or result in atherosclerotic damage if spread to blood [1]. Several studies have found that Insig genetic polymorphisms or deficiency are associated with hypercholesterolaemia. One particularly interesting single nucleotide polymorphism (SNP) which is consistent with either G or C, located 10 kb upstream of *INSIG-2* was reported to have the strongest association with hypercholesterolaemia [86]. Oki et al. demonstrated that the SNP upstream of *INSIG-2* was associated with the prevalence of hypercholesterolaemia but not with obesity in Japanese American women. So, the CC genotype of the SNP is suggested to be a protective genetic factor against the progression of hypercholesterolaemia on a high-fat diet [86].

The deficiency in both Insig-1 and Insig-2 causes the level of HMG-CoA reductase protein to be elevated. Mutant hamster cells that are deficient in Insig-1 but not Insig-2 show partial defects in regulation of reductase degradation and SREBP processing [71]. Another finding demonstrates that Insig deficiency is associated with conditions such as hair and skin defects, facial development and ear structure abnormalities [87], and clefting syndrome in mice [88]. For example, Insig deficiency in skin or an increase in HMG-CoA reductase protein causes the accumulation of cholesterol precursors, and then the hypercholesterolaemia impairs normal hair and skin development. Topical treatment of Epi-Insig-DKO mice with simvastatin or lovastatin, inhibitors of reductase, reduces sterol precursors in skin and corrects the hair and skin defects [87], ameliorates the clefting syndrome [88]. The advances in our understanding of the molecular mechanism of Insigs may ultimately lead to find novel strategies for the treatment of hypercholesterolaemia and other diseases. The other investigations indicate that SREBPs regulate the expression of the LDL receptor which enables the hepatocytes to remove cholesterol contained in LDL particles from the bloodstream. High cholesterol prevents maturation of SREBPs and cuts off cholesterol and LDL receptor synthesis, resulting in high blood cholesterol and the imminent danger of atherosclerotic plaque formation. But drugs which block HMGCoA reductase are the most effective way to interrupt the vicious circle [64], and a new method to treat the syndrome of hypercholesterolaemia.

## Conclusions

We have investigated the functions of peptides in lipid metabolism for many years. Our studies in adipocytes find that peptides such as Obestatin and Ghrelin participate in the regulation of lipometabolism, and Insigs also play an important role in cholesterol metabolism (unpublished data). The cholesterol regulatory system adjusts cholesterol metabolism so as to maintain a constant level of membrane cholesterol. By adjusting these processes, the tissues can acquire additional cholesterol during periods of rapid growth, and they can prevent toxic accumulation of cholesterol when cholesterol is excess. The cellular mechanisms of this cholesterol regulatory system become clearer owning to the discovery of Insigs, but there are still many questions need us to further answer: 1) How does cholesterol-dependent binding of SCAP to Insigs prevent COPII binding? 2) What are the main functional differences between Insig-1 and Insig-2? 3) How do insulin and other factors affect differential regulation of Insigs? 4) How about the functions and the relationship of Insig-2a and Insig-2b? Answers to these questions should reveal new functions of Insigs and their signal transduction mechanism in other scientific areas. Medicinal manipulation of the Insigs-binding system is expected to prove highly beneficial in the management of cholesterol-related disease.

## Abbreviations

ER: Endoplasmic reticulum; HMG-CoA reductase: 3-hydroxy-3-methylglutaryl coenzyme A reductase; Insig: Insuin induced-gene; nSREBP: Nuclear SREB; SCAP: SREBP cleavage-activating protein; SNP: Single nucleotide polymorphism; SREBP: Sterol regulatory element-binding protein; SSD: Sterol-sensing domain.

## Competing interests

The authors declare that they have no competing interests.

## Authors’ contributions

XY Dong and SQ Tang contributed equally to this work, they designed this review, collected data and drafted the manuscript; As the corresponding author of this study, JD Chen participated in design of this review and revised the manuscript. All authors read and approved this version to be published.

## Authors’ information

XY Dong, an associated professor, is involved in fat metabolism and its regulatory mechanism; and SQ Tang had a PhD in Animal Nutrition and mainly studied on peptides and their functions; JD Chen holds a PhD in Preventive Veterinary Medicine and works on immunology and microbiology, he also takes investigation in virus protein and its regulatory functions.
